# Exploring the causes of augmentation in restless legs syndrome

**DOI:** 10.3389/fneur.2023.1160112

**Published:** 2023-09-28

**Authors:** Pengyu Zeng, Tiantian Wang, Lisan Zhang, Fang Guo

**Affiliations:** ^1^Department of Neurobiology, Department of Neurology of Sir Run Run Shaw Hospital, Zhejiang University School of Medicine, Hangzhou, China; ^2^NHC and CAMS Key Laboratory of Medical Neurobiology, MOE Frontier Science Center for Brain Research and Brain-Machine Integration, School of Brain Science and Brain Medicine, Zhejiang University, Hangzhou, China; ^3^Department of Pharmacy, Sir Run Run Shaw Hospital, School of Medicine, Zhejiang University, Hangzhou, China; ^4^Center for Sleep Medicine, Sir Run Run Shaw Hospital, School of Medicine, Zhejiang University, Hangzhou, China

**Keywords:** restless legs syndrome, augmentation, dopamine agonists, iron deficiency, circadian rhythms

## Abstract

Long-term drug treatment for Restless Legs Syndrome (RLS) patients can frequently result in augmentation, which is the deterioration of symptoms with an increased drug dose. The cause of augmentation, especially derived from dopamine therapy, remains elusive. Here, we review recent research and clinical progress on the possible mechanism underlying RLS augmentation. Dysfunction of the dopamine system highly possibly plays a role in the development of RLS augmentation, as dopamine agonists improve desensitization of dopamine receptors, disturb receptor interactions within or outside the dopamine receptor family, and interfere with the natural regulation of dopamine synthesis and release in the neural system. Iron deficiency is also indicated to contribute to RLS augmentation, as low iron levels can affect the function of the dopamine system. Furthermore, genetic risk factors, such as variations in the *BTBD9* and *MEIS1* genes, have been linked to an increased risk of RLS initiation and augmentation. Additionally, circadian rhythm, which controls the sleep–wake cycle, may also contribute to the worsening of RLS symptoms and the development of augmentation. Recently, Vitamin D deficiency has been suggested to be involved in RLS augmentation. Based on these findings, we propose that the progressive reduction of selective receptors, influenced by various pathological factors, reverses the overcompensation of the dopamine intensity promoted by short-term, low-dose dopaminergic therapy in the development of augmentation. More research is needed to uncover a deeper understanding of the mechanisms underlying the RLS symptom and to develop effective RLS augmentation treatments.

## 1. Introduction

Restless legs syndrome, also known as Willis-Ekbom disease (WED), is a sensorimotor disorder characterized by an irresistible urge to move the legs, worsening symptoms at night, and for some patients, unpleasant sensations in the legs. The disorder affects an estimated 1–15.3% of the population, with about 1–3% of people overall experiencing severe and frequent symptoms (1–7). Despite the prevalence of the syndrome, the cause of RLS syndrome remains ambiguous from both clinical and pathophysiological perspectives. Multiple pathological mechanisms, including dysfunction in dopamine-related systems, alterations in adenosine and glutamatergic pathways, brain iron deficiency, and genetic mutations, likely contribute to the etiopathogenesis of RLS (7). The effectiveness of drugs and therapies targeting these mechanisms supports their significant roles in RLS.

Despite the advancements in the development of drug targeting various transmitter pathways, dopaminergic agents(levodopa and dopamine agonists) have remained the mainstay of treatment for restless legs syndrome for the past four decades (7–10). The use of these agents in RLS therapy was initially reported in the 1980s, when Akpinar published a case study documenting the efficacy of levodopa. The long-term efficacy of levodopa and dopaminergic agonists, including pramipexole, ropinirole, and rotigotine, has been well established. Until recent years, they are widely considered the first-line pharmacological treatment for RLS (11–15). However, even with minimal doses of levodopa and dopamine agents, long-term treatment with these drugs leads to a progressive worsening of RLS symptoms (16, 17). This worsening can be differentiated from tolerance, early morning rebound, and the natural progression of RLS or fluctuations in disease severity (18). This deterioration in dopamine therapy, known as augmentation, is the leading cause of treatment discontinuation and failure in RLS (11, 19, 20).

“Augmentation” refers to an iatrogenic exacerbation of RLS symptoms, as characterized by earlier occurrence of symptoms in the afternoon than before treatment initiation, the spread of symptoms to the upper limbs, and a reduced latency until symptoms manifest during periods of rest. Another recognizable characteristic of augmentation is the paradoxical worsening of symptoms upon increasing the dose of dopamine agonists. The clinical phenomenon of augmentation was first observed during long-term levodopa treatment and was suggested to be a result of the treatment itself (21). Augmentation is especially common in patients undergoing levodopa treatment and was initially found in 73% of patient (16). The phenomenon of augmentation is not typically observed in long-term treatment with non-dopamine therapies. If such observations do exist, they are more likely considered a natural progression of RLS rather than an iatrogenic effect (22). However, evaluating the prevalence of augmentation poses challenges due to various influencing factors, including medication type and dosage, study duration and design, assessment criteria, and sample size (11). Specifically, augmentation rates have been found to rise with study duration: short-term studies report rates of 10% (22–26), studies lasting 2–3 years report rates of 15–30%, and studies lasting around 10 years report rates of 42–68% (6, 27, 28). Here, we provide a summary of mechanisms, including dopamine release efficiency, dopamine receptor sensitivity and interaction, genetic risk factors for RLS, iron deficiency, vitamin D deficiency and circadian rhythms. These factors are thought to play a role in the development of augmentation following dopamine agent administration. It can be speculated that the multi-dimensional integration of these pathological elements reverses the relief function of dopamine agents over time.

## 2. Dysfunction of dopamine system in RLS augmentation

### 2.1. The contribution of dopamine inefficiency in RLS augmentation

Several studies have supported the increased dopamine synthesis and secretion on the presynaptic surface in RLS patients (29–32). However, the synaptic concentration of endogenous dopamine and the complex postsynaptic signaling system in augmented RLS patients remains unclear. According to the physiological findings, synaptic dopamine concentrations are primarily influenced by presynaptic D2 and D3 autoreceptors and the dopamine transporter (DAT) (33). D2/D3 agonists lead to increased dopamine and serotonin levels in the prefrontal cortex of rats (34). Chronic administration of pramipexole can lead to desensitization of D3 autoreceptors and reduced dopamine uptake in the mouse striatum, which is apparently comparable to RLS patients (35, 36). Hypotheses suggest that augmentation is also linked to impaired dopamine function in the central nervous system. The therapeutic benefits of dopamine agonists on both sensory and motor symptoms, including periodic limb movement in sleep (PLMS), indicate the role of the dopaminergic system in the pathophysiology of RLS, consistent with a presynaptic hyperdopaminergic state. However, the paradoxical changes of receptor function in mild RLS patients, and potentially in augmented RLS patients, need further investigation.

### 2.2. The contribution of dopamine receptor interactions in RLS augmentation

The interaction between dopamine receptors within the dopamine receptor family and other neurotransmitter systems is implicated in the development of RLS augmentation. Specifically, high dopamine concentrations, possibly resembling the hyperdopaminergic state observed in patients with augmentation, target excitatory dopamine 1 receptors (D1Rs), which sustaining locomotor-like activity in the isolated spinal cord, potentially contributing to augmentation (37). Activation of dopamine 3 receptors (D3Rs) induce both overall sensory and motor excitability in the isolated spinal cord (38, 39), similar to the effect of dopamine agents. Actually, D3Rs and D1Rs form functional heterodimers and heterotetramers (40, 41). Long-term treatment with D3R agonists has also been shown to upregulate excitatory D1Rs in the spinal cord (42). Overall, the downregulation of inhibitory D3Rs and upregulation of excitatory D1Rs seem to contribute to the augmentation of RLS. This propose was partially supported by experiment that long-term treatment with D3R agonists in a rodent model could be reversed or rescued by adjuvant block of D1Rs in animals that no longer responded to the D3R agonist alone (42). The intricate balance of adenosine, dopamine, and glutamate in the striatum may be influenced by the heteromer formed by the combination of D1Rs and dopamine 2 receptors (D2Rs), which interact with adenosine A1 receptors (A1Rs) and A2 receptors (A2ARs), respectively (7). These findings suggest that the dynamic of the pathological network involved in dopamine system in augmented patients may be influenced by internal imbalances of dopamine and extensive neurotransmitter receptors.

### 2.3. Desensitization of dopamine receptors participate in RLS augmentation

Desensitization of dopamine receptors may also contribute to the development of augmentation. Like other G protein-coupled receptors (GPCRs), dopamine receptors regulate signal transduction by receptor desensitization (43). Recent studies revealed that D1 and D2R homo-oligomers undergo desensitization in response to selective agonists (44, 45). In addition, D3Rs are internalized through a mechanism of pharmacological sequestration after agonist-induced activation (46). It is ponderable that desensitization of dopamine receptors may progressively diminish the efficacy of dopamine agonists in augmented RLS patients, despite low-dose administration temporarily enhancing dopamine receptor signaling. This is especially relevant for RLS patients who receive nighttime treatment with dopamine agonists, as dopamine receptor sensitivity is highest during this time (47). It is considered that the treatment of RLS patients with dopamine agonists may induce pathological desensitization of dopamine receptors, resulting in a gradual reduction in the efficacy of these agents. This vulnerability can be confirmed in self-controlled patients undergoing long-term dopamine therapy.

## 3. Iron deficiency may contribute to augmentation

The role of iron in RLS pathogenesis has been established through numerous studies, as well as the positive effect of iron supplementation on RLS symptoms. However, the relationship between iron metabolism and RLS augmentation is still unclear. The severity of RLS symptoms at baseline and with augmentation has been associated with low ferritin levels, which suggests a deficiency in mobilizable iron stores (48, 49). Besides, iron supplementation can provide alleviate and remission of RLS symptoms in some patients (50–52) and prevents or reduces of augmentation during dopaminergic therapy (11). Mechanically, iron deficiency may exacerbate RLS severity through dysregulation of dopamine system. Iron serves as a cofactor for tyrosine hydroxylase, which converts tyrosine to dopamine, and oral iron supplement has been found to reduce dopamine transporter numbers in rat models (53). Iron deficiency is also associated with reduced levels of extracellular dopamine, dopamine transporters, D1Rs, and D2Rs (54). Nonetheless, additional research is required to elucidate the pathological mechanism linking iron deficiency, dopamine dysregulation, and RLS augmentation.

## 4. Genetic risk factors involved in augmentation

The contribution of genetic background to RLS augmentation is poorly understood. The significant physiological and pathological association between genetic risk factors, dopamine dysfunction, and iron metabolism suggests that RLS risk factors contribute to the development of augmentation. The *MEIS1* and *BTBD9* loci have a significant genetic association with RLS (55) and are involved in brain iron metabolism (56). Overexpression of BTBD9 in HEK cells increased ferritin expression in embryonic kidney cells (57). A previous study conducted in Denmark found that the rs9296249 variant in the *BTBD9* gene was significantly associated with serum ferritin levels in female blood donors (58). On the other hand, an association between the *MEIS1* gene and an increase in the expression of H-ferritin, L-ferritin, and divalent metal transporter-1 RNA has been uncovered in the thalamus (59).

*BTBD9* and its homolog are involved in the transcriptional and cellular regulation of dopamine D2Rs and D3Rs, which are the main targets of dopamine agonists. The internalization of D2R is modulated by DNM-1 (60), which showed a significant increase in *BTBD9* systematic knockout mice. The expression of *DNM-1* was found to be enhanced in the *BTBD9* complete knockout mice compared to wild-type mice that were euthanized at midnight (61). The symptoms of RLS patients and the thermal sensory deficit of *BTBD9* complete knockout mice can be alleviated by D2/D3 agonists (62). A research using d*BTBD9* mutant flies showed a significant decrease in brain dopamine levels and an incoordinate sleep phenotype, which was majorly rescued by giving pramipexole (57). In contrast, the increased sensory excitability and locomotor activity in D3KO mice suggest that both sensory and motor circuits are functionally upregulated in the spinal cord of this mouse (63). In both D3KO and iron-deprived mice, a significant increase of the D1R protein expression has been found in the lumbar spinal cord (64, 65). Under *BTBD9* deficiency, the activity of the D1R-mediated dopamine system may have reached its peak, which indicated a weakened D2R signaling but an increased D1R signaling in RLS syndrome (61), similar to augmentation states. In addition, a reasonable viewpoint was that BTBD9 is involved in dopamine biosynthesis by unknown pathways (66). Therefore, RLS patients with risk *BTBD9* loci may have a tendency toward augmentation in the pathological process.

## 5. Vitamin D and RLS augmentation

Vitamin D deficiency has been shown to correlate with RLS symptoms on serum vitamin D level (67, 68). Vitamin D is also associated with pathological factors of RLS, such as iron deficiency (68, 69) and dopamine dysfunction (70, 71). The effect of vitamin D on RLS augmentation has been reported. A case report showed that an 81-year-old woman’s dopaminergic augmentation significantly improved result from the co-treatment involved in vitamin D supplementation (72). Besides, an unpublished study revealed a high prevalence of vitamin D deficiency in 9 female RLS patients using dopaminergic drugs, and these females showed an improved response to vitamin D supplementation (73).

## 6. Circadian rhythms and RLS augmentation

RLS symptoms are more apparent at night, with a higher occurrence reported in the evening and night hours (74). Indeed, a phase advance of anticipated initiation of RLS symptoms, known as a feature of RLS augmentation, was possibly influenced by circadian rhythm. A study indicates that treatment with levodopa advances the dim light melatonin onset (DLMO) in RLS patients experiencing augmentation compared to those without augmentation (75). Moreover, considering the deterioration of RLS symptoms in cases of augmentation, the intensification of augmented symptoms is particularly evident at night. Specifically, unlike akathisia, paresthesias in RLS are typically localized, usually idiopathic, and have a periodic maximum of expression in dusk and night (76). Therefore, given the fluctuation of molecular expression in RLS and the evidence for chronobiotic mechanisms in the disease, the potential role of circadian mechanisms in the RLS deterioration cannot be ignored. (77, 78).

Pathological factors involved in augmentation, such as abnormal dopamine system dynamics, genetic risk factors expression, and iron concentration, display diurnal rhythmicity. This view suggests that the timing of dopamine agonist treatment may influence development of RLS augmentation through pathological networks. Dopamine release has an unambiguous circadian activity pattern of decreasing in the evening and night and increasing in the morning (79–83). In wild-type mice, *D1R*, *D2L*, and *D2S* mRNA levels were all decreased during the locomotion time compared with the sleep time (61). The level of *D2R* mRNA was increased in the striatum of *BTBD9* complete knockout at midnight, indicating that the effect of *BTBD9* deficiency on the *D2R* mRNA level is regulated by circadian rhythm (84). Arrhythmic transcription of dopamine receptor mRNAs may contribute to these findings. Additionally, diurnal variations of iron concentration have been observed in peripheral and regional brain areas (85). Significant diurnal changes in total iron concentrations in brain region were found in mice (86). Diurnal variations of iron-regulated proteins in CSF have been previously described in RLS patients (87). Research on fruit flies has found that the neural circuit connection from the small ventrolateral clock neurons to the protocerebrum are reduced when the iron is chelated from the diet (88). In terms of the genetic factors of RLS, although a study has shown that *BTBD9* mutant mice exhibit abnormal sleep architecture, which is reminiscent of RLS patients (62), it is yet to be determined if *BTBD9* variation impacts circadian. On the other hand, *MEIS1* haploinsufficiency was associated with a sex-dependent increase in activity during the onset of rest, similar to the circadian rhythm of RLS symptoms observed in human patients (89).

## 7. Model of augmentation development

**Figure 1:**
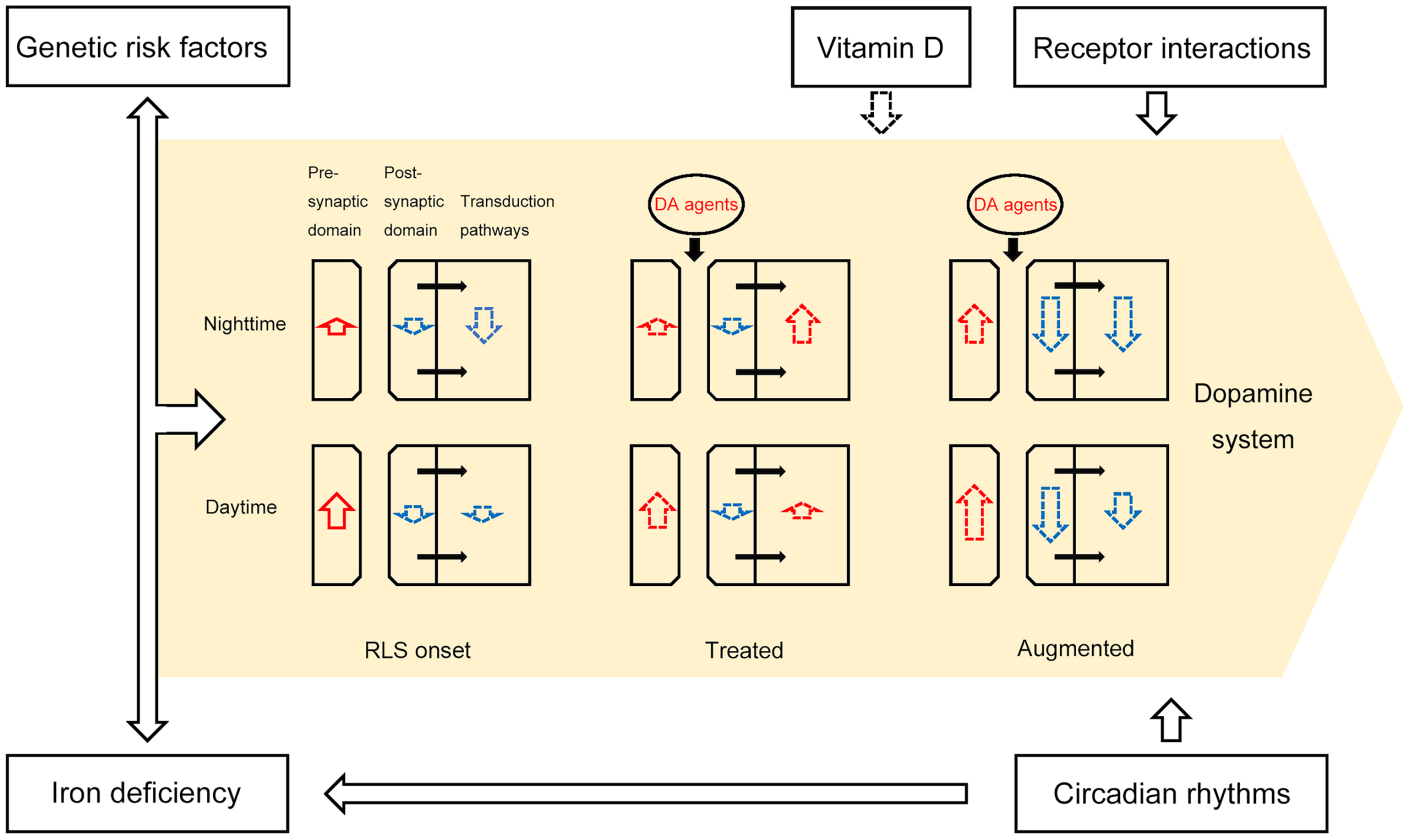
A schematic depicting the pathological model of RLS augmentation. The intensities of upregulation (length of red arrows) and downregulation (length of blue arrows) in presynaptic and postsynaptic DA signaling, as well as the signal strength of DAR downstream transduction pathways, changed during RLS augmentation. The black arrows within the yellow region represent biological processes between the post-synaptic domain and transduction pathways. Iron deficiency, genetic risk factors, receptor interactions, vitamin D, and circadian rhythms contribute to dopamine system dysfunction, as depicted by the black arrows outside the yellow region. Dopaminergic agents such as levodopa, pramipexole, ropinirole, and rotigotine temporarily increase the overall effect of dopamine on postsynaptic receptors, but irreversibly reduce the number of dopamine receptors. Dotted black or colored arrows indicate potential relationships between two entities.

Overall, the effect of dopamine agents, potentially accompanied with iron deficiency, genetic risk factors, vitamin D deficiency and effect of circadian rhythms, contributes to the attenuation of dopamine signaling in patients with augmented RLS. RLS patients may exhibit impaired postsynaptic response to increased dopamine stimulation after long-term administration of low-dose dopamine agents, possibly due to dopamine receptor desensitization and interaction of dopamine receptors. When dopamine signaling is reduced during dusk and night, this insufficient postsynaptic dopamine signaling causes augmented RLS symptoms. More specifically, despite an overall increase in dopamine secretion in RLS patients, continuous treatment with dopamine agents may desensitize selective dopamine receptors, with a gradual decrease in peak dopamine efficiency over time. This leads to a deficit in dopamine signaling in the evening and at night compared with healthy people. As a result, the circadian attenuation of dopamine signaling in the evening and at night in augmented RLS patients disrupts normal sleep homeostasis, with the symptom of fragmented sleep at rest in these patients. The dopamine drug-induced incremental activation of dopamine signaling exacerbates the downregulation of overall post-synaptic dopamine receptors, thus worsening the RLS disease and expanding the symptoms (Figure 1).

## 8. Conclusion

Augmentation is one of the greatest threats to RLS patients in the context of the widespread usage of dopamine agents. The pathological mechanism behind this symptom is unclear and needs to be elucidated. Current clinical diagnoses and research findings highlight the potential mechanisms linking dopamine dysfunction, circadian regulation, iron deficiency, vitamin D deficiency and genetic risk factors to the development of augmentation. However, a multi-perspective investigation in the field is lacking. In addition, the role of other transmitter signaling pathways in the development of augmentation remains vague. Further careful and long-term research is necessary to fully understand the underlying mechanisms of augmentation in RLS patients.

## Author contributions

PZ contributed to the original concept of the article, did the literature search, wrote the original version of the manuscript, drew the figure and reviewed it as it progressed. TW, LZ, and FG contributed to the original concept of the manuscript and reviewed the manuscript. All authors contributed to the article and approved the submitted version.

## References

[1] Migueis DP Lopes MC Casella E Soares PV Soster L Spruyt K. Attention deficit hyperactivity disorder and restless leg syndrome across the lifespan: a systematic review and meta-analysis. Sleep Med Rev. (2023) 69:101770. doi: 10.1016/j.smrv.2023.101770 36924608

[2] Allen RP Walters AS Montplaisir J Hening W Myers A Bell TJ. Restless legs syndrome prevalence and impact: REST general population study. Arch Intern Med. (2005) 165:1286–92. doi: 10.1001/archinte.165.11.1286 15956009

[3] Cho S-J Hong JP Hahm B-J Jeon HJ Chang SM Cho MJ. Restless legs syndrome in a community sample of Korean adults: prevalence, impact on quality of life, and association with DSM-IV psychiatric disorders. Sleep. (2009) 32:1069–76. doi: 10.1093/sleep/32.8.1069 19725258 PMC2717197

[4] Trenkwalder C Paulus W. Restless legs syndrome: pathophysiology, clinical presentation and management. Nat Rev Neurol. (2010) 6:337–46. doi: 10.1038/nrneurol.2010.55 20531433

[5] Yilmaz K Kilincaslan A Aydin N Kor D. Prevalence and correlates of restless legs syndrome in adolescents. Dev Med Child Neurol. (2011) 53:40–7. doi: 10.1111/j.1469-8749.2010.03796.x 20875044

[6] Wang T Ying M Zhao R Zhu D Zhang L. Augmentation in patients with restless legs syndrome receiving pramipexole therapy: a retrospective study in a single center from China. Sleep Breath. (2022) 26:373–80. doi: 10.1007/s11325-021-02353-9 33864178

[7] Manconi M Garcia-Borreguero D Schormair B Videnovic A Berger K Ferri R. Restless legs syndrome. Nat Rev Dis Primers. (2021) 7:80. doi: 10.1038/s41572-021-00311-z 34732752

[8] Garcia-Borreguero D Cano-Pumarega I. New concepts in the management of restless legs syndrome. BMJ. (2017) 356:j104. doi: 10.1136/bmj.j104 28242627

[9] Scholz H Trenkwalder C Kohnen R Riemann D Kriston L Hornyak M. Dopamine agonists for restless legs syndrome. Cochrane Database Syst Rev. (2011) 2011:CD006009. doi: 10.1002/14651858.CD006009.pub2 21412893 PMC8908466

[10] Winkelman JW Armstrong MJ Allen RP Chaudhuri KR Ondo W Trenkwalder C. Practice guideline summary: treatment of restless legs syndrome in adults: report of the guideline development, dissemination, and implementation Subcommittee of the American Academy of neurology. Neurology. (2016) 87:2585–93. doi: 10.1212/WNL.0000000000003388 27856776 PMC5206998

[11] Garcia-Borreguero D Silber MH Winkelman JW Högl B Bainbridge J Buchfuhrer M. Guidelines for the first-line treatment of restless legs syndrome/Willis–Ekbom disease, prevention and treatment of dopaminergic augmentation: a combined task force of the IRLSSG, EURLSSG, and the RLS-foundation. Sleep Med. (2016) 21:1–11. doi: 10.1016/j.sleep.2016.01.017 27448465

[12] Fleishon H Mandel S Arenas A. Anterior spinal artery syndrome after cervical injection of heroin. Arch Neurol. (1982) 39:739. doi: 10.1001/archneur.1982.00510230065026 7126007

[13] Garcia-Borreguero D Grunstein R Sridhar G Dreykluft T Montagna P Dom R. A 52-week open-label study of the long-term safety of ropinirole in patients with restless legs syndrome. Sleep Med. (2007) 8:742–52. doi: 10.1016/j.sleep.2006.09.009 17512789

[14] Montplaisir J Karrasch J Haan J Volc D. Ropinirole is effective in the long-term management of restless legs syndrome: a randomized controlled trial. Mov Disord. (2006) 21:1627–35. doi: 10.1002/mds.21050 16874755

[15] Silber MH Ehrenberg BL Allen RP Buchfuhrer MJ Earley CJ Hening WA. An algorithm for the Management of Restless Legs Syndrome. Mayo Clin Proc. (2004) 79:916–22. doi: 10.4065/79.7.916 15244390

[16] Silber MH Buchfuhrer MJ Earley CJ Koo BB Manconi M Winkelman JW. The Management of Restless Legs Syndrome: an updated algorithm. Mayo Clin Proc. (2021) 96:1921–37. doi: 10.1016/j.mayocp.2020.12.026 34218864

[17] Trenkwalder C Paulus W. Pharmacological treatments of augmentation in restless legs syndrome patients. Adv Pharmacol. (2019) 84:255–65. doi: 10.1016/bs.apha.2019.02.002 31229175

[18] García-Borreguero D. Dopaminergic augmentation in restless legs syndrome/Willis-Ekbom disease: identification and management. Sleep Med Clin. (2015):10:287–292, xiii. doi: 10.1016/j.jsmc.2015.05.020 26329438

[19] Garcia-Borreguero D Cano-Pumarega I Marulanda R. Management of treatment failure in restless legs syndrome (Willis-Ekbom disease). Sleep Med Rev. (2018) 41:50–60. doi: 10.1016/j.smrv.2018.01.001 29602660

[20] Khachatryan SG Ferri R Fulda S Garcia-Borreguero D Manconi M Muntean M-L. Restless legs syndrome: over 50 years of European contribution. J Sleep Res. (2022) 31:e13632. doi: 10.1111/jsr.13632 35808955 PMC9542244

[21] García-Borreguero D Williams A-M. Dopaminergic augmentation of restless legs syndrome. Sleep Med Rev. (2010) 14:339–46. doi: 10.1016/j.smrv.2009.11.006 20219397

[22] Allen RP Chen C Garcia-Borreguero D Polo O DuBrava S Miceli J. Comparison of Pregabalin with Pramipexole for restless legs syndrome. N Engl J Med. (2014) 370:621–31. doi: 10.1056/NEJMoa1303646 24521108

[23] Allen RP Ondo WG Ball E Calloway MO Manjunath R Higbie RL. Restless legs syndrome (RLS) augmentation associated with dopamine agonist and levodopa usage in a community sample. Sleep Med. (2011) 12:431–9. doi: 10.1016/j.sleep.2011.03.003 21493132

[24] García-Borreguero D Högl B Ferini-Strambi L Winkelman J Hill-Zabala C Asgharian A. Systematic evaluation of augmentation during treatment with ropinirole in restless legs syndrome (Willis-Ekbom disease): results from a prospective, multicenter study over 66 weeks. Mov Disord. (2012) 27:277–83. doi: 10.1002/mds.24889 22328464

[25] Högl B Garcia-Borreguero D Trenkwalder C Ferini-Strambi L Hening W Poewe W. Efficacy and augmentation during 6months of double-blind pramipexole for restless legs syndrome. Sleep Med. (2011) 12:351–60. doi: 10.1016/j.sleep.2010.12.007 21354368

[26] Oertel WH Benes H Garcia-Borreguero D Geisler P Högl B Trenkwalder C. One year open-label safety and efficacy trial with rotigotine transdermal patch in moderate to severe idiopathic restless legs syndrome. Sleep Med. (2008) 9:865–73. doi: 10.1016/j.sleep.2008.04.012 18753003

[27] Garcia-Borreguero D Cano-Pumarega I Garcia Malo C Cruz Velarde JA Granizo JJ Wanner V. Reduced response to gabapentin enacarbil in restless legs syndrome following long-term dopaminergic treatment. Sleep Med. (2019) 55:74–80. doi: 10.1016/j.sleep.2018.11.025 30772697

[28] Silver N Allen RP Senerth J Earley CJ. A 10-year, longitudinal assessment of dopamine agonists and methadone in the treatment of restless legs syndrome. Sleep Med. (2011) 12:440–4. doi: 10.1016/j.sleep.2010.11.002 21239226

[29] Earley CJ Hyland K Allen RP. CSF dopamine, serotonin, and biopterin metabolites in patients with restless legs syndrome. Mov Disord. (2001) 16:144–9. doi: 10.1002/1531-8257(200101)16:1<144::AID-MDS1009>3.0.CO;2-F 11215576

[30] Earley CJ Connor J Garcia-Borreguero D Jenner P Winkelman J Zee PC. Altered brain iron homeostasis and dopaminergic function in restless legs syndrome (Willis-Ekbom disease). Sleep Med. (2014) 15:1288–301. doi: 10.1016/j.sleep.2014.05.009 25201131

[31] Connor JR Wang X-S Allen RP Beard JL Wiesinger JA Felt BT. Altered dopaminergic profile in the putamen and substantia nigra in restless leg syndrome. Brain. (2009) 132:2403–12. doi: 10.1093/brain/awp125 19467991 PMC2732265

[32] Salminen AV Clemens S García-Borreguero D Ghorayeb I Li Y Manconi M. Consensus guidelines on the construct validity of rodent models of restless legs syndrome. Dis Model Mech. (2022) 15:dmm049615. doi: 10.1242/dmm.049615 35946581 PMC9393041

[33] Chang P-K Chien K-Y Chen J-C. Dopamine transporter is downregulated and its association with chaperone protein Hsc70 is enhanced by activation of dopamine D3 receptor. Brain Res Bull. (2020) 165:263–71. doi: 10.1016/j.brainresbull.2020.10.005 33049353

[34] Chernoloz O El Mansari M Blier P. Long-term administration of the dopamine D3/2 receptor agonist pramipexole increases dopamine and serotonin neurotransmission in the male rat forebrain. J Psychiatry Neurosci. (2012) 37:113–21. doi: 10.1503/jpn.110038 22023785 PMC3297071

[35] Castro-Hernández J Afonso-Oramas D Cruz-Muros I Salas-Hernández J Barroso-Chinea P Moratalla R. Prolonged treatment with pramipexole promotes physical interaction of striatal dopamine D3 autoreceptors with dopamine transporters to reduce dopamine uptake. Neurobiol Dis. (2015) 74:325–35. doi: 10.1016/j.nbd.2014.12.007 25511804

[36] Luis-Ravelo D Fumagallo-Reading F Castro-Hernandez J Barroso-Chinea P Afonso-Oramas D Febles-Casquero A. Prolonged dopamine D3 receptor stimulation promotes dopamine transporter ubiquitination and degradation through a PKC-dependent mechanism. Pharmacol Res. (2021) 165:105434. doi: 10.1016/j.phrs.2021.105434 33484816

[37] Allen RP. Restless leg syndrome/Willis-Ekbom disease pathophysiology. Sleep Med Clin. (2015) 10:207–14. doi: 10.1016/j.jsmc.2015.05.022 26329430 PMC4559751

[38] Keeler BE Baran CA Brewer KL Clemens S. Increased excitability of spinal pain reflexes and altered frequency-dependent modulation in the dopamine D3-receptor knockout mouse. Exp Neurol. (2012) 238:273–83. doi: 10.1016/j.expneurol.2012.09.002 22995602

[39] Sharples SA Humphreys JM Jensen AM Dhoopar S Delaloye N Clemens S. Dopaminergic modulation of locomotor network activity in the neonatal mouse spinal cord. J Neurophysiol. (2015) 113:2500–10. doi: 10.1152/jn.00849.2014 25652925 PMC4416552

[40] Cruz-Trujillo R Avalos-Fuentes A Rangel-Barajas C Paz-Bermúdez F Sierra A Escartín-Perez E. D3 dopamine receptors interact with dopamine D1 but not D4 receptors in the GABAergic terminals of the SNr of the rat. Neuropharmacology. (2013) 67:370–8. doi: 10.1016/j.neuropharm.2012.11.032 23238327

[41] Guitart X Navarro G Moreno E Yano H Cai N-S Sánchez-Soto M. Functional selectivity of allosteric interactions within G protein–coupled receptor oligomers: the dopamine D1-D3 receptor Heterotetramer. Mol Pharmacol. (2014) 86:417–29. doi: 10.1124/mol.114.093096 25097189 PMC4164978

[42] Dinkins M-L Lallemand P Clemens S. Long-term treatment with dopamine D3 receptor agonists induces a behavioral switch that can be rescued by blocking the dopamine D1 receptor. Sleep Med. (2017) 40:47–52. doi: 10.1016/j.sleep.2017.10.001 29221778

[43] Gainetdinov RR Premont RT Bohn LM Lefkowitz RJ Caron MG. Desensitization of G protein-coupled receptors and neuronal functions. Annu Rev Neurosci. (2004) 27:107–44. doi: 10.1146/annurev.neuro.27.070203.144206 15217328

[44] Lamey M Thompson M Varghese G Chi H Sawzdargo M George SR. Distinct residues in the carboxyl tail mediate agonist-induced desensitization and internalization of the human dopamine D1 receptor. J Biol Chem. (2002) 277:9415–21. doi: 10.1074/jbc.M111811200 11773080

[45] Ng GY Varghese G Chung HT Trogadis J Seeman P O’Dowd BF. Resistance of the dopamine D2L receptor to desensitization accompanies the up-regulation of receptors on to the surface of Sf9 cells. Endocrinology. (1997) 138:4199–206. doi: 10.1210/endo.138.10.5433 9322930

[46] Xu W Reith MEA Liu-Chen L-Y Kortagere S. Biased signaling agonist of dopamine D3 receptor induces receptor internalization independent of β-arrestin recruitment. Pharmacol Res. (2019) 143:48–57. doi: 10.1016/j.phrs.2019.03.003 30844536

[47] Garcia-Borreguero D Larrosa O Granizo JJ de la Llave Y Hening WA. Circadian variation in neuroendocrine response to L-dopa in patients with restless legs syndrome. Sleep. (2004) 27:669–73. doi: 10.1093/sleep/27.4.669 15283001

[48] Çurgunlu A Döventaş A Karadeniz D Erdinçler DS Oztürk AK Karter Y. Prevalence and characteristics of restless legs syndrome (RLS) in the elderly and the relation of serum ferritin levels with disease severity: hospital-based study from Istanbul, Turkey. Arch Gerontol Geriatr. (2012) 55:73–6. doi: 10.1016/j.archger.2011.06.002 21722973

[49] Trenkwalder C Högl B Benes H Kohnen R. Augmentation in restless legs syndrome is associated with low ferritin. Sleep Med. (2008) 9:572–4. doi: 10.1016/j.sleep.2007.07.020 17921065

[50] Avni T Reich S Lev N Gafter-Gvili A. Iron supplementation for restless legs syndrome - a systematic review and meta-analysis. Eur J Intern Med. (2019) 63:34–41. doi: 10.1016/j.ejim.2019.02.009 30798983

[51] Bae H Cho YW Kim KT Allen RP Earley CJ. Randomized, placebo-controlled trial of ferric carboxymaltose in restless legs syndrome patients with iron deficiency anemia. Sleep Med. (2021) 84:179–86. doi: 10.1016/j.sleep.2021.05.036 34157632

[52] Yang X Yang B Ming M Li S Wang F Zhu Z. Efficacy and tolerability of intravenous iron for patients with restless legs syndrome: evidence from randomized trials and observational studies. Sleep Med. (2019) 61:110–7. doi: 10.1016/j.sleep.2019.01.040 31395522

[53] LaBarbera V Trotti LM Rye D. Restless legs syndrome with augmentation successfully treated with IV iron. Neurol Clin Pract. (2017) 7:e26–8. doi: 10.1212/CPJ.0000000000000276 28680768 PMC5490383

[54] Dauvilliers Y Winkelmann J. Restless legs syndrome: update on pathogenesis. Curr Opin Pulm Med. (2013) 19:594–600. doi: 10.1097/MCP.0b013e328365ab07 24048084

[55] Schormair B Zhao C Bell S Tilch E Salminen AV Pütz B. Identification of novel risk loci for restless legs syndrome in genome-wide association studies in individuals of European ancestry: a meta-analysis. Lancet Neurol. (2017) 16:898–907. doi: 10.1016/S1474-4422(17)30327-7 29029846 PMC5755468

[56] Gonzalez-Latapi P Malkani R. Update on restless legs syndrome: from mechanisms to treatment. Curr Neurol Neurosci Rep. (2019) 19:54. doi: 10.1007/s11910-019-0965-4 31250128

[57] Freeman A Pranski E Miller RD Radmard S Bernhard D Jinnah HA. Sleep fragmentation and motor restlessness in a Drosophila model of restless legs syndrome. Curr Biol. (2012) 22:1142–8. doi: 10.1016/j.cub.2012.04.027 22658601 PMC3381864

[58] Sørensen E Grau K Berg T Simonsen AC Magnussen K Erikstrup C. A genetic risk factor for low serum ferritin levels in Danish blood donors. Transfusion. (2012) 52:2585–9. doi: 10.1111/j.1537-2995.2012.03629.x 22486183

[59] Catoire H Dion PA Xiong L Amari M Gaudet R Girard SL. Restless legs syndrome-associated MEIS1 risk variant influences iron homeostasis. Ann Neurol. (2011) 70:170–5. doi: 10.1002/ana.22435 21710629

[60] Iwata K Ito K Fukuzaki A Inaki K Haga T. Dynamin and rab5 regulate GRK2-dependent internalization of dopamine D2 receptors. Eur J Biochem. (1999) 263:596–602. doi: 10.1046/j.1432-1327.1999.00549.x 10406971

[61] Lyu S Doroodchi A Xing H Sheng Y DeAndrade MP Yang Y. BTBD9 and dopaminergic dysfunction in the pathogenesis of restless legs syndrome. Brain Struct Funct. (2020) 225:1743–60. doi: 10.1007/s00429-020-02090-x 32468214 PMC7429108

[62] DeAndrade MP Johnson RL Unger EL Zhang L van Groen T Gamble KL. Motor restlessness, sleep disturbances, thermal sensory alterations and elevated serum iron levels in Btbd9 mutant mice. Hum Mol Genet. (2012) 21:3984–92. doi: 10.1093/hmg/dds221 22678064 PMC3428151

[63] Meneely S Dinkins M-L Kassai M Lyu S Liu Y Lin C-T. Differential dopamine D1 and D3 receptor modulation and expression in the spinal cord of two mouse models of restless legs syndrome. Front Behav Neurosci. (2018) 12:199. doi: 10.3389/fnbeh.2018.00199 30233336 PMC6131574

[64] Brewer KL Baran CA Whitfield BR Jensen AM Clemens S. Dopamine D3 receptor dysfunction prevents anti-nociceptive effects of morphine in the spinal cord. Front Neural Circuits. (2014) 8:62. doi: 10.3389/fncir.2014.00062 24966815 PMC4052813

[65] Zhao H Zhu W Pan T Xie W Zhang A Ondo WG. Spinal cord dopamine receptor expression and function in mice with 6-OHDA lesion of the a11 nucleus and dietary iron deprivation. J Neurosci Res. (2007) 85:1065–76. doi: 10.1002/jnr.21207 17342757

[66] Shaw PJ Duntley SP. Neurological disorders: towards a mechanistic understanding of restless legs syndrome. Curr Biol. (2012) 22:R485–6. doi: 10.1016/j.cub.2012.05.004 22720681 PMC3741047

[67] Wali S Shukr A Boudal A Alsaiari A Krayem A. The effect of vitamin D supplements on the severity of restless legs syndrome. Sleep Breath. (2015) 19:579–83. doi: 10.1007/s11325-014-1049-y 25148866

[68] Wali S Alsafadi S Abaalkhail B Ramadan I Abulhamail B Kousa M. The association between vitamin D level and restless legs syndrome: a population-based case-control study. J Clin Sleep Med. (2018) 14:557–64. doi: 10.5664/jcsm.7044 29609719 PMC5886433

[69] Smith EM Tangpricha V. Vitamin D and anemia: insights into an emerging association. Curr Opin Endocrinol Diabetes Obes. (2015) 22:432–8. doi: 10.1097/MED.0000000000000199 26414080 PMC4659411

[70] Baksi SN Hughes MJ. Chronic vitamin D deficiency in the weanling rat alters catecholamine metabolism in the cortex. Brain Res. (1982) 242:387–90. doi: 10.1016/0006-8993(82)90331-6 6288172

[71] Oran M Unsal C Albayrak Y Tulubas F Oguz K Avci O. Possible association between vitamin D deficiency and restless legs syndrome. Neuropsychiatr Dis Treat. (2014) 10:953–8. doi: 10.2147/NDT.S63599 24899811 PMC4039397

[72] Department of Neurology, Near East University School of Medicine, Nicosia, Cyprus, Diker S. Restless leg syndrome: role of Iron and vitamin D deficiencies. Cyprus. J Med Sci. (2018):114–6. doi: 10.5152/cjms.2018.347

[73] Cederberg KLJ Silvestri R Walters AS. Vitamin D and restless legs syndrome: a review of current literature. Tremor Other Hyperkinet Mov (N Y). (2023) 13:12. doi: 10.5334/tohm.741 37034443 PMC10077981

[74] Garcia Borreguero D Winkelmann J Allen RP. Introduction: towards a better understanding of the science of RLS/WED. Sleep Med. (2017) 31:1–2. doi: 10.1016/j.sleep.2016.10.007 27894926

[75] Garcia-Borreguero D Serrano C Larrosa O Granizo JJ. Circadian effects of dopaminergic treatment in restless legs syndrome. Sleep Med. (2004) 5:413–20. doi: 10.1016/j.sleep.2004.01.007 15223002

[76] Hening WA. Restless legs syndrome: a sensorimotor disorder of sleep/wake motor regulation. Curr Neurol Neurosci Rep. (2002) 2:186–96. doi: 10.1007/s11910-002-0029-y 11898486

[77] Garcia-Borreguero D Larrosa O de la Llave Y. Circadian aspects in the pathophysiology of the restless legs syndrome. Sleep Med. (2002) 3:S17–21. doi: 10.1016/s1389-9457(02)00143-0 14592162

[78] Michaud M Dumont M Selmaoui B Paquet J Livia Fantini M Montplaisir J. Circadian rhythm of restless legs syndrome: relationship with biological markers. Ann Neurol. (2004) 55:372–80. doi: 10.1002/ana.10843 14991815

[79] Chung S Lee EJ Yun S Choe HK Park S-B Son HJ. Impact of circadian nuclear receptor REV-ERBα on midbrain dopamine production and mood regulation. Cells. (2014) 157:858–68. doi: 10.1016/j.cell.2014.03.039 24813609

[80] Earley CJ Hyland K Allen RP. Circadian changes in CSF dopaminergic measures in restless legs syndrome. Sleep Med. (2006) 7:263–8. doi: 10.1016/j.sleep.2005.09.006 16564215

[81] Khaldy H León J Escames G Bikjdaouene L García JJ Acuña-Castroviejo D. Circadian rhythms of dopamine and dihydroxyphenyl acetic acid in the mouse striatum: effects of pinealectomy and of melatonin treatment. Neuroendocrinology. (2002) 75:201–8. doi: 10.1159/000048238 11914592

[82] Liang X Holy TE Taghert PH. Polyphasic circadian neural circuits drive differential activities in multiple downstream rhythmic centers (2022) 33:351–63. doi: 10.1101/2022.10.11.511837 PMC9877191 36610393

[83] Tang Q Assali DR Güler AD Steele AD. Dopamine systems and biological rhythms: Let’s get a move on. Front Integr Neurosci. (2022) 16:957193. doi: 10.3389/fnint.2022.957193 35965599 PMC9364481

[84] Akhisaroglu M Kurtuncu M Manev H Uz T. Diurnal rhythms in quinpirole-induced locomotor behaviors and striatal D2/D3 receptor levels in mice. Pharmacol Biochem Behav. (2005) 80:371–7. doi: 10.1016/j.pbb.2004.11.016 15740778

[85] Unger EL Earley CJ Beard JL. Diurnal cycle influences peripheral and brain iron levels in mice. J Appl Physiol. ((1985) (2009)) 106:187–93. doi: 10.1152/japplphysiol.91076.2008 PMC2636939 18988764

[86] Unger EL Jones BC Bianco LE Allen RP Earley CJ. Diurnal variations in brain iron concentrations in BXD RI mice. Neuroscience. (2014) 263:54–9. doi: 10.1016/j.neuroscience.2013.12.056 24406439 PMC3977075

[87] Earley CJ Ponnuru P Wang X Patton SM Conner JR Beard JL. Altered iron metabolism in lymphocytes from subjects with restless legs syndrome. Sleep. (2008) 31:847–52. doi: 10.1093/sleep/31.6.847 18548829 PMC2442411

[88] Rudisill SS Martin BR Mankowski KM Tessier CR. Iron deficiency reduces synapse formation in the Drosophila clock circuit. Biol Trace Elem Res. (2019) 189:241–50. doi: 10.1007/s12011-018-1442-7 30022428 PMC6338522

[89] Salminen AV Garrett L Schormair B Rozman J Giesert F Niedermeier KM. Meis1: effects on motor phenotypes and the sensorimotor system in mice. Dis Model Mech. (2017) 10:981–91. doi: 10.1242/dmm.030080 28645892 PMC5560065

